# Biomedical Publishing Metrics as a Barrier to Knowledge Dissemination

**DOI:** 10.7759/cureus.115765

**Published:** 2026-09-04

**Authors:** Brian W Rotenberg, Douglas Chepeha

**Affiliations:** 1 Otolaryngology, Western University, London, CAN; 2 Otolaryngology - Head and Neck Surgery, University of Michigan, Ann Arbor, USA

**Keywords:** altmetric score, citation index, eigenfactor, journal impact factor, publishing metrics, pubmed indexed research

## Abstract

The Journal Impact Factor (JIF) is an outdated biomedical metric that hinders knowledge dissemination. Relying on citation counts creates a skewed, easily manipulated system with a temporal lag that ignores global equity. Frontline clinicians consume research to improve care but rarely generate citations, meaning the JIF cannot capture real-world clinical utility. To ensure rankings reflect true value, publishers must adopt a multi-dimensional matrix prioritizing usage-based Altmetrics, such as verified downloads and views, to instantly measure global clinical application.

## Editorial

Biomedical journals have long relied on a single proxy to define the prestige and quality of scientific discovery: the Journal Impact Factor (JIF). Originally conceived in the 1960s as a tool to help librarians optimize their subscription budgets, the JIF has evolved into the single most used metric to assign value to a biomedical journal. Yet, in an era defined more by open science, digital dissemination, and urgent global health challenges, assessing the structural worth of medical journals through a solitary metric is anachronistic.

The problems of the JIF include its susceptibility to strategic manipulation, its lack of actual clinical utility, and its friction with open-access publishing models [1]. If the biomedical publishing world is going to truly reflect how novel scientific insights shape clinical medicine, publishers must move past citation counts and adopt a framework that values real-time application and knowledge equity.

The JIF is calculated as the average number of citations earned each year by items published during the preceding two-year window. This formula treats citation distribution as something that is statistically normally distributed. However, citation behavior can be highly skewed. A tiny minority of exceptionally high-yielding papers can mathematically inflate the numerator of a journal, creating a false impression that a median paper within that same periodical offers equivalent scientific value [2]. That is rarely the case.

This calculation structure invites systemic distortions. Editorial boards seeking to artificially elevate their standing may engage in "citation engineering," subtly pressuring authors into self-citation or strategically clustering broad review articles, which naturally amass higher citation volumes, to the start of a publication cycle. This can shift the journal's focus away from substantive scientific merit and toward gaming the metric system itself [3].

More critically, the traditional citation network operates with a severe temporal lag. When a breakthrough occurs in therapy or diagnosis, clinicians need to rapidly translate that insight into active protocols. However, the traditional peer-review and subsequent publication pipeline can mean that a newly published paper may require as much as one to two years to generate a recognizable citation footprint [4]. Consequently, the JIF is structurally incapable of capturing real-time responses to epidemics or public health crises.

The global transition toward Open Access models exposes a conflict between legacy metrics and global health equity. Under historic paywalled subscription models, top-tier journals restricted cutting-edge literature to affluent academic medical centers or individual paying subscribers. Conversely, modern open science demands that clinical insights be accessible to frontline practitioners, independent physicians, and researchers operating in low- and middle-income nations. When access barriers are removed, a stark divergence emerges between academic citation and practical dissemination.

For example, a clinical study detailing a novel pediatric triage protocol in resource-limited settings might be downloaded tens of thousands of times by frontline medical officers, directly reducing regional infant mortality rates. Yet, because these active practitioners focus exclusively on patient care rather than authoring academic literature, that same critical study may barely yield even one formal citation. By over-relying on the JIF, the academic hierarchy systematically undervalues journals publishing pragmatic globally accessible research, favoring instead niche, theoretical bench science written by and for an insular ecosystem of publishing academics.

To align biomedical journal rankings with how medical literature is actually consumed, we recommend formal elevation of usage-based Altmetrics, specifically verified full-text downloads and unique abstract views, to a status that matches or exceeds citation records like JIF [1]. Modern data architectures, such as the audited Counting Online Usage of Networked Electronic Resources (COUNTER) standards, provide tamper-resistant protocols capable of tracking exact digital consumption patterns without vulnerability to manipulation.

First, download and view velocities register immediately upon publication. During the initial stages of a public health crisis, tracking daily full-text downloads of epidemiological or therapeutic preprints highlights which journals the medical workforce are actually reading long before those works clear the multi-year backlog of formal citation indexing. Second, the overwhelming majority of medical readers consist of practicing clinicians, epidemiologists, policymakers, and health educators. These professionals consume literature to refine patient care and shape institutional guidelines, not to write and cite papers. Usage statistics capture this vast, real-world clinical value that traditional citations completely erase. Third, while legacy citation networks are prone to systemic inequities, universal download data levels the playing field by directly measuring a paper’s intrinsic clinical appeal and utility, which in turn reflects on the journal that published it.

Moving away from a reliance on the JIF does not mean stepping into an unregulated digital wilderness. Instead, it requires the adoption of a diversified, multi-dimensional evaluation matrix [5]. Evaluation platforms must implement composite scorecards that balance academic citation with broader societal dissemination. This multi-pronged framework should actively track audited HTML/PDF download velocities, data repository re-use rates, and formal integration into major policy documents. By deploying these composite indices, the scientific community can ensure that a journal’s ranking actually reflects its dual purpose: driving the absolute cutting edge of theoretical discovery while simultaneously serving as an active, real-time engine of global clinical practice.

The JIF remains a legacy analog tool attempting to navigate an intricate, digital, and increasingly open-access medical ecosystem. Continuing to anchor medical excellence to this narrow, easily gamed, and delayed metric can obscure where true clinical progress is occurring and in which journals. We recommend that academic medical institutions, funding bodies, and premier publishers formally de-emphasize the JIF in favor of Altmetrics. Embracing transparent, real-time consumption data will bring biomedical journal evaluation systems into alignment with how we want care for our patients: rapidly, equitably, and based entirely on real-world impact.
